# Language as a Threat: Multimodal Evaluation and Interventions for Overwhelming Linguistic Anxiety in Severe Aphasia

**DOI:** 10.3389/fpsyg.2019.00678

**Published:** 2019-05-08

**Authors:** María José Torres-Prioris, Diana López-Barroso, José Paredes-Pacheco, Núria Roé-Vellvé, Marc S. Dawid-Milner, Marcelo L. Berthier

**Affiliations:** ^1^Cognitive Neurology and Aphasia Unit (UNCA), Centro de Investigaciones Médico-Sanitarias, Instituto de Investigación Biomédica de Málaga – IBIMA, University of Málaga, Málaga, Spain; ^2^Area of Psychobiology, Faculty of Psychology, University of Málaga, Málaga, Spain; ^3^Molecular Imaging Unit, Centro de Investigaciones Médico-Sanitarias, General Foundation of the University of Malaga, Málaga, Spain; ^4^Molecular Imaging and Medical Physics Group, Department of Psychiatry, Radiology and Public Health, University of Compostela, Santiago de Compostela, Spain; ^5^Biomedical Research Networking Center in Bioengineering, Biomaterials and Nanomedicine (CIBER-BBN), Barcelona, Spain; ^6^Neurophysiology of Autonomic Nervous System Laboratory, Centro de Investigaciones Médico-Sanitarias, University of Málaga, Málaga, Spain

**Keywords:** linguistic anxiety, stress, aphasia, language assessment, autonomic response, neuroimage, Rotigotine

## Abstract

Linguistic anxiety (LA) is an abnormal stress response induced by situations that require the use of verbal behavior, and it is accentuated during language testing in persons with aphasia (PWA). The presence of LA in PWA may jeopardize the interpretation of cognitive evaluations, leading to biased conclusions about the severity of the language alteration and the effectiveness of the treatments. In the present study, we report the case of a woman (Mrs. A) with severe chronic mixed transcortical aphasia due to left frontal and parietal hemorrhages that partially spared the perisylvian area. Mrs. A was treated with the dopamine agonist Rotigotine alone and combined with Intensive Language-Action Therapy (ILAT). Complementary evaluations included autonomic reactivity during the performance of different language tasks, resting state functional magnetic resonance imaging (rs-fMRI) and [18F]-fluorodeoxyglucose positron emission tomography (18F-FDG-PET). We found that formal language testing in a clinical setting triggered a dramatic increase of automatic echolalia, perseverations and frustration, making the task completion difficult. The treatment improved aphasia, but gains were more robust when evaluation was performed by Mrs. A’s husband at home than by clinicians. Autonomic evaluation under Rotigotine revealed higher reactivity during tasks tapping an impaired function in comparison with a task evaluating a preserved function (verbal repetition). Baseline 18F-FDG-PET analysis showed decreased metabolic activity in left limbic-paralimbic areas, whereas rs-fMRI revealed compensatory activity in the right hemisphere. We also analyzed the different factors (e.g., premorbid personality traits, task difficulty) that may have contributed to LA in Mrs. A during language testing. Our findings emphasize the usefulness of implicating adequately trained laypersons in the evaluation and treatment of PWA showing LA. Further studies using multidimensional evaluations are needed to disentangle the interplay between anxiety and abnormal language as well as the neural mechanisms underpinning LA in PWA.

## Introduction

Aphasia is a neuropsychological syndrome characterized by an impairment of language, affecting speech production (spontaneous speech, naming and repetition) and auditory comprehension as well as the ability to read and write. Aphasic syndromes are frequently classified upon performance on verbal repetition tasks (Albert et al., 1981). In accordance, aphasias are broadly classified in two groups: (1) aphasias characterized by impaired verbal repetition, the so-called *perisylvian aphasias* (e.g., Broca’s and Wernickes aphasias); and (2) those with preserved verbal repetition, named *transcortical aphasias*. Aphasia mainly occurs as a consequence of brain damage in the left hemisphere, usually due to stroke (Hillis, 2007). Previous research has evidenced that stroke is commonly associated to pervasive emotional changes (e.g., depression, anxiety, irritability, apathy, impaired awareness) (Hackett et al., 2014; Babulal et al., 2015; Robinson and Jorge, 2016; Døli et al., 2017). These psychological and behavioral alterations may be a consequence of focal and distant brain changes caused by the lesion which might disrupt brain networks implicated in the control of mood and behavior (House, 1987; Castillo et al., 1993; Beblo et al., 1999; Chemerinski and Robinson, 2000). But emotional changes may also be linked to adjustment problems related to the imposed life changes resulting from cognitive and motor deficits (Chemerinski and Robinson, 2000). Further, factors such as personality traits (Aben et al., 2002; Greenop et al., 2009), coping strategies (Vuger-Kovačić et al., 2007), family support (Morris et al., 1991) and premorbid mood disorders (Burvill et al., 1995; Sembi et al., 1998; Leppävuori et al., 2003) may also influence outcomes. All this suggests the need to adopt a multidimensional approach to identify the multiple factors that contribute to the emotional and psychological consequences of post-stroke aphasia.

Among the above-mentioned, anxiety is one of the most frequent emotional consequences of stroke (Fure et al., 2006). The prevalence of anxiety in stroke victims was estimated to be between 20 and 24% (Burton et al., 2013). However, this figure may be misrepresented since screening tools for anxiety disorders are not always suitable for persons with aphasia (PWA), a reason that explains why this stroke population is frequently excluded from anxiety studies (Burton et al., 2013). Recently, Morris et al. (2017) reported that 44% of PWA have significant levels of anxiety, as revealed by questionnaires filled by the patients’ relatives, being this prevalence higher than the one found in the general stroke population (20–24%) (Burton et al., 2013; Shehata et al., 2015). Another study, based in self-reports of a sample of stroke patients, found no significant differences in anxiety prevalence between an aphasic group and a non-aphasic group (Døli et al., 2017). However, the authors did find than higher anxiety level (i.e., more reported symptoms) was associated with impaired repetition and comprehension deficits (Døli et al., 2017).

An important area of concern and enquiry among neuropsychologists and speech pathologists working with PWA is how anxiety levels affect linguistic performance and how this may interfere with clinical decision making. The term “linguistic anxiety” (LA) has been coined to describe the stress reactions induced by language testing and everyday situations that require the use of language in PWA (Cahana-Amitay et al., 2011). The inability of PWA to use language to communicate effectively may precipitate an excessive emotional reactions (i.e., anxiety state, frustration, irritability) during language testing, the so-called “catastrophic reaction” (Goldstein, 1948; Starkstein et al., 1993). Thus, the anticipation of committing verbal errors or the inability to perform a given task may be perceived as a stressful situation that triggers frustration and anger (Goldstein, 1948). This means that in clinical settings, the existing language deficits might be reinforced by the anxiety reaction which may lead to inaccurate conclusions about the clinical profile and severity of aphasia and the effectiveness of therapeutic interventions. Intriguingly, despite the relevance and potential clinical implications of LA, very few studies have addressed its impact on language performance in the aphasic population (Laures-Gore et al., 2007; Cahana-Amitay et al., 2015; Laures-Gore and Buchanan, 2015). In addition, there is limited evidence on the contributing factors (e.g., premorbid personality traits, lesion location) and the behavioral, psychological and physiological characteristics of anxiety response in PWA. Relevantly, it is still unclear whether brain damaged persons will have similar physiological response as healthy subjects exposed to anxiogenic events, and how lesions affecting areas involved in “higher” modulation of the autonomic response may alter this reaction.

The paucity of reports on this issue stimulates performing exploratory studies that help to design protocols with an integrative approach suitable even for persons with severe aphasia (e.g., global aphasia, mixed transcortical aphasia [MTCA]). Herein, we report the case of an elderly woman (Mrs. A) with severe chronic post-stroke MTCA who displayed an overwhelming LA during language evaluation when this was performed by the therapist, but not when a trained naïve relative performed the evaluation and treatment. Therefore, the aims of the present study were: (a) to describe the LA profile of Mrs. A highlighting its clinical implications; (b) to explore Mrs. A’s autonomic responses during testing of different language functions; and (c) to characterize the associated structural and functional brain correlates of LA using multimodal neuroimaging.

Before providing details on the case of Mrs. A, we analyze the current state of LA in aphasia.

## Framework for Studies on Linguistic Anxiety

Persons with aphasia logically show greater perceived stress than healthy subjects during linguistic tasks (Laures-Gore et al., 2007), and this stress response may interfere with their linguistic performance. Multiple variables may modulate the subjective emotional response triggered when the PWA is confronted with situations that need to be solved using language. Laures-Gore and Buchanan (2015) proposed a multifactorial framework for the study of LA in aphasia positing that the stress response in a given language-situation is influenced by different individual factors. In the present study, we focused on two individual factors that we suggest have special relevance in the causality of LA in Mrs. A. First, we consider the role of premorbid personality traits such as excessive perfectionism and high self-criticism. These obsessional traits may interact with other individual factors (i.e., expectation of improvement, awareness of failures) by self-imposing a high performance to comply with the demands of language testing, and ultimately potentiating the LA response. Second, we consider the role that direct and remote (e.g., due to diaschisis) effects of the lesion may play in both the subjective emotional appraisal of the situation (i.e., language testing) and the related physiological response. Lastly, we also take into account other factors that may influence the emergence of LA and that are related to the context of evaluation and the difficulty of the tasks required to perform, in direct relation with the profile and severity of the aphasia. In the next section we review the mentioned factors to provide evidence on their contribution to the response of LA. Similarly, we also review the characteristics of the anxiety response both in healthy subjects and the existing evidence in subjects with brain damage.

### Individual Factors Influencing Anxiety Response

Premorbid personality traits, self-efficacy, aphasia severity, and brain areas affected by the lesion may be of special importance in the emergence of LA. In some cases, post-stroke anxiety may not only be the consequence of brain damage (Robinson and Starkstein, 1990), or be linked to the imposed life changes, but it may also reflect premorbid mood profiles or disorders. Burvill et al. (1995) found that 33.3% of men and 50% of women with post-stroke anxiety disorders showed evidence of either depression or anxiety before the stroke onset (see also Sembi et al., 1998; Leppävuori et al., 2003 for similar data on this issue, and Burton et al., 2013 for a review). Thus, it seems reasonable to suggest that some premorbid individual characteristics, such as an obsessional personality style, might have an important role in triggering anxiety in PWA. Obsessional traits include perfectionism, disproportionate preoccupation for details, order and organization, and excessive devotion to work and productivity (American Psychiatric Association, 2013). High comorbidity of obsessional traits, obsessive-compulsive disorder and other anxiety disorders have been reported (Pena-Garijo et al., 2013)^1^.

Further, the interaction between low self-efficacy - defined as the perceived (in)ability to perform a given task (Bandura, 1982) - and high levels of anxiety has been widely studied in the psychology field (Bandura, 1988). Studies of foreign language learning suggest that low self-efficacy negatively correlates with the level of LA, and this relationship determines language achievements (Cheng et al., 1999; Chen and Lin, 2009). In this vein, it seems obvious that the presence of a severe aphasia may itself favor the perception of low self-efficacy in language performance, at least in subjects aware of their deficits, increasing the likelihood of LA. However, since self-efficacy is a subjective judgment, LA is also present in mild aphasias (Cahana-Amitay et al., 2015).

Another individual factor that may influence the expression of LA in PWA is the location and extension of the lesion. Anxiety triggers a hyperactivation of the sympatho-adrenomedullary (SA) and the hypothalamic-pituitary-adrenocortical axes (HPA) (Ulrich-Lai and Herman, 2009), a response regulated by cortical and subcortical brain mechanisms. Overlapping circuits in the limbic forebrain, the hypothalamus, and the brainstem are the main components of the physiological response to stress as they can generate a rapid reaction to re-establish and maintain the homeostatic status through direct connections with the SA and HPA (Ulrich-Lai and Herman, 2009). The role of limbic and paralimbic cortices such as amygdala, hippocampus (HC), medial prefrontal cortex (mPFC), anterior cingulate cortex (ACC), and orbitofrontal cortex (OFC) is related to the subjective appraisal of immediate and potential stressors and the high-order regulation of the physiological response via subcortical connections (Ulrich-Lai and Herman, 2009; Shin and Liberzon, 2010; Fiddick, 2011).

Thus, the cortical-subcortical interplay seems to be crucial for the emotional regulation of anxiety. Anatomical and functional differences together with changes in this network induced by local or remote effects of a lesion may explain interindividual differences in the level of perceived anxiety and the resultant physiological response (Critchley et al., 2003; Buchanan et al., 2009, 2010; Hahn et al., 2011; Makovac et al., 2016). Like this, the disruption of the fronto-amygdalar network has been proposed as a fingerprint of anxiety disorders. Decreased resting state functional connectivity (rs-fMRI) between the amygdala and the OFC has been identified in social anxiety disorders (Hahn et al., 2011). Additionally, autonomic response in brain damaged patients may be reduced due to disruption in the above-mentioned network (Buchanan et al., 2010).

### Task and Context Related Factors Influencing the Anxiety Response

Clinical contexts may induce an excessive stress response in many subjects. In the medical field, this phenomenon is known as the “white coat effect” and it refers to the autonomic reactivity induced by the mere presence of a physician (Pickering et al., 2002). Nevertheless, neuropsychological assessment may even be a more powerful stress-inducing situation since it also involves direct testing. Anxiety elicited by a testing situation has been associated with lower cognitive performance (e.g., in working memory tasks) (Coy et al., 2011), so that language evaluation by professionals in PWA can be viewed by as a potential stressor. In addition, a factor directly related to LA response is the difficulty of the task, which is also related to the individual profile of aphasia. For instance, speech production tasks would imply more difficulty in a person with non-fluent aphasia (e.g., Broca’s aphasia), than in cases of fluent aphasia with impaired and reduced awareness of language deficits.

### Linguistic Anxiety Response: Physiological Correlates and Linguistic Performance

Previous studies in healthy subjects suggest that anxiety response is characterized by an increase in the respiration rate, sympathetic activation [increased heart rate (HR), electrodermal activity and blood pressure] (Kreibig, 2010), and cortisol levels (Laures-Gore et al., 2007). It remains to be determined, however, whether the autonomic correlates of anxiety in healthy subjects and in PWA are the same, and which specific factors may influence the physiological response to stressful situations in PWA. Few studies on this topic have been reported up to now. Cahana-Amitay et al. (2015) reported increased HR during a speech preparation task in comparison to a non-linguistic task in a person with mild aphasia, but no prominent changes were observed in electrodermal activity (EDA). Further, a study of a person with acquired apraxia of speech reported increased systolic and diastolic blood pressure when speech therapy was administered by a clinician (stressful context) in comparison to a conditions where the patient self-directed the same treatment (Sakamoto et al., 1999), thus suggesting that persons with speech disorders are highly susceptible to stress induced by clinical settings. In a study of cortisol reactivity, Laures-Gore et al. (2007) found that the linguistic task did not induce changes in cortisol level, although the participants reported higher perceived stress than in a control non-linguistic task. Nonetheless, in a later study these authors did find cortisol reactivity induced by a speech production task in PWA (Laures-Gore et al., 2010).

Importantly, LA in PWA has psychological and behavioral consequences that may negatively impact speech performance interfering with recovery. For instance, in the abovementioned study Cahana-Amitay et al. (2015) showed that autonomic reactivity was accompanied by lower discourse productivity in a PWA. In healthy subjects, public speaking tasks induce autonomic changes that are related to reduced speech fluency (i.e., higher pause time) (Buchanan et al., 2014). More evidence on the detrimental effect of anxiety in language performance comes from studies that examined the impact of anxiety in the performance of a foreign/non-mastered language. Significant negative relationship between second language performance and anxiety is frequently reported in a variety of language domains such as speaking (Phillips, 1992; Aida, 1994; Woodrow, 2006), listening (Zhang, 2013), reading (Saito et al., 1999), and writing (Cheng et al., 1999).

## Materials and Methods

### Case Description

**FIGURE 1:**
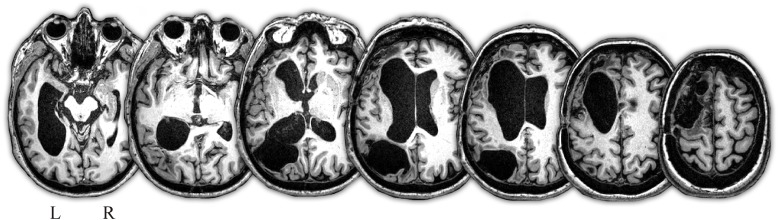
T1-weighted MRI axial images depicting extensive brain damage in the left hemisphere. There are two old cavitated lesions, one involving the left dorsolateral and medial frontal cortex with partial compromise of the anterior perisylvian language area (inferior frontal gyrus, pars triangularis), whereas the other lesion involved the left inferior parietal lobe (angular gyrus) and medial occipital lobe. There are also marked asymmetric dilatation of the lateral ventricle especially of the left frontal and temporal horns. Note that great part of the left perisylvian language core is spared by the lesions and no damage was observed in the right hemisphere. Images are shown in neurological convention. L, left; R, right.

Mrs. A was a 66-years-old right-handed woman who suffered a stroke the day of her professional retirement, causing her two simultaneous hemorrhagic lesions that affected frontal and parietal areas in the left hemisphere, partially sparing the core perisylvian language areas (see Figure 1). Mrs. A was a monolingual native Spanish speaker with normal language development during childhood. She was a highly educated woman (24 years of formal education), previously working as a full professor of mathematics at a Spanish University. Mrs. A was referred to our unit for aphasia evaluation 27 months post-onset. At that moment, she had a mild right hemiparesis, right visual field defect and a severe aphasia. Mrs. A had a severe non-fluent aphasia characterized by markedly reduced spontaneous speech and almost nil auditory comprehension in the face of preserved language repetition (MTCA) (Berthier, 1999). Note that this type of aphasia is equivalent to global aphasia, except for the preservation of repetition capacity. Automatic echolalia, verbal perseverations, and ready-made expressions were very frequent in Mrs. A. Further, she could sing overlearned songs without prompting using excellent lyrics and melody.

Several episodes of LA were detected in Mrs. A throughout the language evaluations. During language testing sessions she would frequently get frustrated and when she was not able to perform a linguistic task, she blocked out and reiteratively said “I don’t know, I don’t know, I don’t know…”. After that, the evaluation had to be stopped, jumping to the next task since she refused to continue with that task. In other occasions, when she was blocked out with an item that she did not understand or was not able to name, she would go back to the items, even after several items had passed, and tried to get feedback (on the name or meaning) from the therapist. This was usually accompanied by face expressions and body gestures indicating worry and tension. We observed that during testing her verbal behavior was replete with echolalic emissions, which markedly increased when she was anxious and upset. However, Mrs. A’s husband reported that echolalia was not excessively pronounced in other contexts (e.g., home, interactions with family members). The presence of severe LA and worsening of language performance when Mrs. A was assessed in a clinical setting by a therapist jeopardized the interpretation of cognitive evaluations, leading to biased conclusions about the severity of the language alteration and the effectiveness of the treatments. However, when language evaluation was performed by Mrs. A’s husband her performance improved significantly.

### Study Design

**FIGURE 2:**
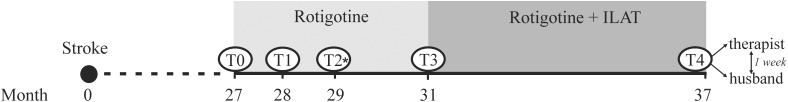
A schematic diagram of the pharmacological treatment and aphasia therapy shows baseline and different type points under treatment with Rotigotine alone and Rotigotine combined with Intensive Language-Action Therapy (ILAT). ^∗^The autonomic study was performed in T2. In T4, the language assessment was divided in two parts. First, the therapist performed the first assessment, and 1 week after, the language evaluation was performed by Mrs. A’s husband.

Mrs. A underwent 5 formal language evaluations in our unit over a 1-year period (see Figure 2). Baseline evaluation (T0) was completed 27 months after stroke. Thereafter, she began pharmacological treatment with Rotigotine (transdermal patches) alone, a drug that modulates the dopaminergic system (Gorgoraptis et al., 2012). Mrs. A initially received Rotigotine alone (without language therapy) for 4 months. Three language evaluations were performed during this period [month 28 (T1); month 29 (T2); and month 31 (T3)]. She also received treatment with oral Domperidone to prevent nausea and vomiting. Assessment at T2 consisted of two parts. In the first part, Mrs. A underwent a formal language evaluation conducted by one therapist (the same therapist that assessed her at T0 and T1) in a quiet room. In the second part, Mrs. A was invited to move to an adjacent unit where she was evaluated by a different therapist while her autonomic response was monitored. Three persons were present at the time of this latest evaluation, one physician who registered the autonomic activity and two therapists who assessed and recorded Mrs. A’s verbal responses during three different language tasks.

Thereafter, treatment with Rotigotine was combined with aphasia therapy using the Spanish version of an Intensive Language-Action therapy (ILAT) (Pulvermüller and Berthier, 2008; Difrancesco et al., 2012; Berthier et al., 2014b). Mrs. A underwent a final evaluation 6 months after combined therapy (Rotigotine plus ILAT) [month 37 (T4)]. At this endpoint, we conducted two independent testing sessions. First, a language battery was administered by an experienced therapist (same therapist that have evaluated her in the previous time points) and, 1 week later, the same battery was administered by Mrs. A’s husband (see *Language Assessment and Aphasia Therapy Administration by a Relative*).

### Pharmacological Treatment

Mrs. A was treated with the non-ergoline dopamine agonist Rotigotine (with high affinity for D1, D2, and D3 receptors) to augment her markedly reduced speech production and other language deficits (see Gill and Leff, 2014). Rotigotine was selected over more traditional ergoline dopaminergic agents, like bromocriptine, because it has better tolerance and adverse event profile (cardiac valve fibrosis). She started with a dose of 2 mg/day for 1 week and then the dose was gradually tapered at a rate of 2 mg per week up to 8 mg/day.

At present, Rotigotine is approved for the treatment of Parkinson’s disease and restless legs syndrome (Benitez et al., 2014). However, given the favorable safety and efficacy profiles of Rotigotine found in stroke patients (Gorgoraptis et al., 2012; Zarola, 2018), the senior author (MLB), a board-certified neurologist with experience in the pharmacological treatment of post-stroke aphasia (see Berthier, 2005) considered that the prescription of this agent for an unapproved use (post-stroke aphasia) was appropriate for this particular case (Wittich et al., 2012). Moreover, since post-stroke MTCA like the one presented by Mrs. A is an exceptional syndrome (4 out of in 1.200 cases – 0.33%) (Bogousslavsky et al., 1988), the most viable option was to treat her in a clinical setting. Taking this into consideration, ethics approval by an ethic committee was not required in accordance with applicable institutional and national guidelines and regulations. Therefore, Rotigotine was used according to the statement of ethical principles for medical research involving human subjects of the Declaration of Helsinki (section 37: Unproven Interventions in Clinical Practice). Mrs. A and her husband were provided with the package leaflet of Rotigotine and they were also fully informed about the pharmacological characteristics, potential benefits and adverse events of the drug and neuroimaging (18-FDG-PET and MRI) acquisition. They were also fully informed about all the procedure across the study (language evaluations, autonomic recording, neuroimaging). Mrs A and her husband agreed to receive the treatment. Written informed consent for research participation in this study as well as for the publication of the case report was obtained from Mrs. A and from Mrs. A’s husband. During both the titration phase (4 weeks) and whole drug treatment he was contacted to detect potential adverse event and evaluate the adherence to the drug.

### Language Therapy

The ILAT is a language therapy based on three principles that includes: (1) intensive practice, (2) use of language action games to introduce a meaningful communication setting; and (3) promotion of the use of verbal language, avoiding non-verbal communication. It requires massive practice over a short period of time (3 h per day for 2 weeks) and guidance by modeling. Although it is normally applied in small groups as a therapeutic game context (2 or 3 participants and a therapist), it can also be applied in a dyad (participant and therapist). Participants are required to communicate verbally in order to ask for picture cards to each other (or to the therapist if the therapy is implemented in dyad). For that, they might name or describe the depicted pictures, as well as comprehend requests made by others. The ILAT comprises 1.100 picture cards of 6 different categories and frequencies (nouns, minimal pairs, colors, numbers, adjectives and actions) divided in two identical pairs of 550 cards (see methodology of administration in Difrancesco et al., 2012). In the present case, the therapy was administered in a dyad by a naïve person, Mrs. A’s husband, who was previously trained in the application of ILAT.

### Neuropsychological Testing

Baseline evaluation testing (T0) included a detailed evaluation of language and other cognitive functions related to right hemisphere functioning (see Table 1). The Aphasia Quotient (AQ) of the Western Aphasia Battery (WAB) was used to rate aphasia severity (Kertesz, 1982; Kertesz et al., 1990) across all evaluations. The WAB is a tool to assess linguistic functioning, including the domains of spontaneous speech (fluency and information content), naming, auditory-verbal comprehension, and verbal repetition of words and phrases. For further language testing, selected subtests of the Psycholinguistic Assessments of Language Processing in Aphasia (PALPA) (Kay et al., 1992; Valle and Cuetos, 1995), targeting verbal repetition and reading domains were used. Additional verbal repetition tasks with different linguistic stimuli were also used (see Table 1).

**Table 1:** Neuropsychological baseline evaluation.

Language assessment	Score	Normative data
***Western Aphasia Battery (WAB)***		
**Aphasia Quotient (AQ) (max = 100)**	**26**	99.6 ± 0.3^a,b^
**Spontaneous Speech (max = 20)**	**0**	20^b^
Information Content (max = 10)	0	
Fluency (max = 10)	0	
**Auditory Verbal Comprehension (max = 10)**	**2.1**	10^b^
Yes/No Question (max = 60)	33	
Auditory Word Recognition (max = 60)	9	
Sequential Commands (max = 80)	0	
**Repetition (max = 10)**	**9.2**	9.9 ± 0.1^b^
**Naming and Word Finding (max = 10)**	**1.7**	9.8 ± 0.1^b^
Object Naming (max = 60)	15	
Word Fluency (max = 20)	0	
Sentences Completion (max = 10)	2	
Responsive Speech (max = 10)	0	
***Sentences completion (max = 40)***	**9**	39.3 ± 0.6^c^

***Verbal Repetition Tests***		
**PALPA 9. Repetition: Imageability × Frequency (max = 160)**	135	
*Word repetition* (max = 80)	80	78.77 ± 1.93^b^
*Non-word repetition* (max = 80)	**55**	77.68 ± 3.35^b^
**Word triplets (high-frequency)**		
*Random combination* (max = 20)	20	19 ± 0.8^c^
*Constrained information* (max = 20)	20	19.4 ± 0.6^c^
**Word triplets (low-frequency)**		
*Random combination* (max = 20)	18	17 ± 2.5^c^
*Constrained information* (max = 20)	20	18.7 ± 1.2^c^
**Idiomatic clichés (max = 40)**	**35**	39.45 ± 0.9^c^
**Novel sentences (max = 40)**	**36**	38.75 ± 1.1^c^

***Reading***		
**PALPA 29. Imageability × Frequency (max = 80)**	**73**	79.53 ± 0.55^b^
**PALPA 30. Grammatical class (max = 80)**	**61**	79.53 ± 0.42^b^
**PALPA 31. Grammatical class and Imageability (max = 40)**	**33**	39.91 ± 0.29^b^
**PALPA 32. Morphology (max = 30)**^d^	**25**	29.61 ± 0.46^b^
**PALPA 33. Regularity** (**max = 60)**	**43**	55.9 ± 5.84^b^
**PALPA 34. Non-words (max = 24)**	**6**	23.22 ± 0.94^b^

**Right Hemisphere Testing**		**Classification (Percentile rank)**

**Raven’s Colored Progressive Matrices**	26	Bellow average^e^
**Visual Form Discrimination Test**^f^	30	Superior (86+)
**Facial Recognition test**^f^	48	High average (72–85)


*^a^Patients with an Aphasia Quotient score under 97.3 are considered to be aphasics. ^*b*^Normative data was extracted from their reference manuals, i.e., WAB subtests from the revised version of the Western Aphasia Battery (Kertesz, 2007); and PALPA subtests from the Spanish version of PALPA (i.e., EPLA) (Valle and Cuetos, 1995) ^*c*^Normative data was extracted from Berthier (2001). ^*d*^Only 30 out of the 60 stimuli included in this subtest were used. ^*e*^Normative score based on patient’s age and education level is 32.8 (±3.9) (Measso et al., 2009). ^*f*^Test from Benton (1994). Scores below the normative data are highlighted in bold.*

In the following evaluations (T1, first part of T2, T3, and T4), only the WAB was administered to Mrs. A (see Table 2). For the second part of T2, consisting in language assessment while autonomic response was acquired, three subtests of the WAB were used, two of them targeting auditory-verbal comprehension (Yes/No Questions (YNQ), Auditory Word Recognition (AWR); and one targeting Verbal Repetition (VR)] (see the *Autonomic Response Monitoring During Language Assessment*).

**Table 2:** Results from language testing across evaluations.

	BL	Rotigotine Treatment		
		
	T0	T1	T2	T3
**Western Aphasia Battery (WAB)**				
**Aphasia Quotient (AQ) (max = 100)**	26	32.6	33.5	34.2
***Spontaneous Speech* (max = 20)**	0	2	3	3
Information Content (max = 10)	0	2	3	3
Fluency (max = 10)	0	0	0	0
***Auditory Verbal Comprehension* (max = 10)**	2.1	3.1	2.75	2.9
Yes/No Question (max = 60)	33	51	42	42
Auditory Word Recognition (max = 60)	9	11	13	16
Sequential Commands (max = 80)	0	0	0	0
***Repetition* (max = 10)**	9.2	10	9.8	9.4
***Naming and Word Finding* (max = 10)**	1.7	1.2	1.2	1.8
Object Naming (max = 60)	15	10	8	12
Word Fluency (max = 20)	0	0	0	0
Sentences Completion (max = 10)	2	2	4	4
Responsive Speech (max = 10)	0	0	0	0

	**Rotigotine + Speech Therapy**			
	
	**T4**			
	
	**Therapist Evaluation**		**Relative Evaluation**	

**Western Aphasia Battery (WAB)**				
**Aphasia Quotient (AQ) (max = 100)**	35.3		47.4	
***Spontaneous Speech* (max = 20)**	3		7	
Information Content (max = 10)	3		4	
Fluency (max = 10)	0		3	
***Auditory Verbal Comprehension* (max = 10)**	3.35		3.4	
Yes/No Question (max = 60)	42		42	
Auditory Word Recognition (max = 60)	25		26	
Sequential Commands (max = 80)	0		0	
***Repetition* (max = 10)**	10		10	
***Naming and Word Finding* (max = 10)**	1.3		3.3	
Object Naming (max = 60)	11		21	
Word Fluency (max = 20)	0		2	
Sentences Completion (max = 10)	2		6	
Responsive Speech (max = 10)	0		4	


*BL, Baseline*.

### Neuropsychiatric Testing

The Leyton Obsessional Inventory (LOI) (Cooper, 1970) was used to identify whether Mrs. A had premorbid obsessive-compulsive symptoms and traits (e.g., perfectionist, and self-criticism). The LOI is a self-rated scale that quantifies the range of obsessional thoughts and compulsive behaviors. It contains 69 “yes/no” questions dealing with the subjective assessment of obsessional symptoms (questions 1–46) and traits (questions 47–69) as well as the degree of resistance and interference with the patient’s life. Since Mrs. A had a severe aphasia, the LOI was completed by her husband and son independently to ascertain agreement and disagreement in the type and number of responses. Both subjects were instructed to complete the LOI by taken in consideration the premorbid symptoms and traits of Mrs. A and not the potential changes in her behavior developed after the stroke. The LOI also contains Resistance and Interference scales (Cooper, 1970) which evaluate symptom severity. These two scales were no administered to Mrs. A’s husband and son because they cannot rate the degree of interference in daily life caused by obsessions and compulsions as well as her effort to resist obsessive thoughts and compulsions. Moreover, based on the behavior of Mrs. A during language testing, the therapist completed the Catastrophic Reaction Scale (CRS) (Starkstein et al., 1993). This is an 11-item scale based on symptoms and signs traditionally described in patients with CR including anxiety, fear, tearfulness, sadness, angry manner, swearing, displacement, refusals, hopelessness, and boasting. It was specially devised to rate the presence and severity of CR in stroke patients. Scores range from 0 (no symptoms) to 3 (severe symptoms) and scores above 8 indicate the presence of CR. These two scales were not tested during follow-up evaluations.

### Language Assessment and Aphasia Therapy Administration by a Relative

As abovementioned, it was noted that Mrs. A became anxious and frustrated during language testing. Therefore, therapists realized that she was not a good candidate to be evaluated and treated by a clinician and thence it was decided to train her husband in the evaluation of aphasia with the WAB (Kertesz, 1982; Kertesz et al., 1990) and in the administration of the ILAT (Difrancesco et al., 2012). This last decision was made because there is evidence that ILAT can be efficiently accomplished by trained laypersons with results comparable to that of experienced therapists (Meinzer et al., 2007). Aphasia therapy was administered by Mrs. A’s husband after T3. He was a retired full professor of mathematics and he was deeply implicated with the treatment of Mrs. A. Every time they visited our Unit for a follow-up, he brought notes with his every-day observations referred to the verbal and communicative behavior showed by Mrs. A, highlighting day-to-day changes in her language and behavior. Before receiving training with ILAT, he had spent about 1 h every day with Mrs. A training naming and writing skills since the stroke onset.

Mrs. A’s husband was provided with the ILAT materials (manual of administration, set of cards, and registration sheets) (Berthier et al., 2014b). He read the manual at home and during the following week, one of us explained the therapy and trained him through role-playing. Once he felt comfortable with the therapy, he started to apply it in a dyad to Mrs. A in a daily basis, 6 days/week (3 h/day). We also asked him to record fragments of the therapy administration 2 time/week for the first 2 weeks in order to provide him feedback of the execution, and after that telephonic interviews were regularly made with him to resolve any question related to the application of ILAT. He also was trained in the use of the WAB. First, general information about the test was given, and then we pointed important aspects to consider in the application of each subtest. He was instructed to allow her wife time to answer, without giving phonological or semantic cues except for the object naming task, just when enough time to answer was given. Also, he was informed to prevent her wife for lip-reading in the repetition task as well as how to place the elements (pen, booklet, and comb) used in the Sequential Commands subtest. At the time he administered the WAB he recorded the whole application. WAB correction was done by two therapists independently. The final score was reach by agreement between the two therapists.

### Autonomic Response Monitoring During Language Assessment

In order to have a measure of the impact of language assessment in the physiological arousal of the Mrs. A, different autonomic responses were monitored while she was required to perform the three selected subtests of the WAB. The recorded autonomic variables included: electrodermal activity (EDA), electrocardiogram (ECG) and instantaneous heart rate (HR), mean arterial blood pressure (ABP), systolic blood pressure (SBP), and diastolic blood pressure (DBP). ECG and HR were recorded in DII. The corresponding devices for monitoring ABP and EDA were placed at the left arm. Autonomic variables were acquired using the Biopac MP150 equipped with an electrocardiographic amplifier SG100B and EDA response amplifier GSR100B. Continuous beat-to-beat arterial blood pressure was registered with bmeyer’s Nexfin. EDA was used as an index of implicit emotional state that may emerge beyond explicit intent. All variables were analyzed using AcqKnowledge software version 5 (Biopac Systems, Inc.). Evaluation was carried out in a quiet room of the Lab of Autonomic Nervous System. Mrs. A was seated at a comfortable armchair with her left arm placed at the armrest, and was instructed to be relaxed, attending to what the therapist was going to ask her and to do not move the left arm during the evaluation. Before starting testing session, a 10-min rest period was introduced while listening to calm music. Baseline autonomic measures were registered at this period. After this baseline period, the three subtests of the WAB were administered in the following order: (1) YNQ, (2) AWR, and (3) VR. The administration of each test was separated by a 3-min rest period.

The selection of the WAB subtests was based on the difficulty that they represented for the patient by considering her performance in previous evaluation as well as the profile and severity of aphasia. The aim was to compare the autonomic response triggered by highly demanding linguistic tasks against a low demanding task and rest (baseline). As high-demanding tasks an auditory comprehension task that required verbal responses (YNQ), and an auditory comprehension task that required motor responses (AWR) were selected. As a low-demanding task, a repetition task composed of word, numbers and phrases (VR) was selected. Other production subtests of the WAB (naming, verbal fluency) could not be used to evaluate autonomic responses because the patient performed at floor and could have led her to disengage from the task, as previously suggested (Laures-Gore et al., 2010). It was hypothesized that the autonomic reactivity would be more evident in the two tasks in which Mrs. A performed worse (high language demand) than in the task in which she showed a nearly perfect execution (low language demand). YNQ and AWR stimuli were presented every 30 s, while VR items were presented every 15 s. This was done because Mrs. A’s verbal repetition was fully preserved and, hence, she could automatically repeat any verbal stimulus (words, numbers and phrases) immediately (during the first 2 or 3 s) after the auditory presentation. On the other hand, since the YNQ and AWR entailed more difficulty, longer time was needed to answer.

AcqKnowledge software version 5 (Biopac Systems, Inc.) was used to extract autonomic data. First, each channel, reflecting each of the autonomic variables, was visually inspected for artifacts. In very few cases artifact coincided with the time after stimulus presentation (time in which the patient was requested to give an answer). In those cases, data for that item had to be discarded. Second, autonomic data for each stimulus was manually extracted. For all three tasks, the average and the maximum and minimum levels of each measure was calculated for each event (time interval between the end of a given stimulus and the beginning of the next one). In the YNQ and AWR tasks, values (average, maximum and minimum) were determined in the 0 – 30 s time interval after the presentation of the stimulus, whereas in the VR task the values were calculated in the 0 – 15 s time interval after the stimulus presentation. To get baseline measures that can be statistically compared to the tasks, the 10 min baseline recording was fragmented in events of 30 s. Afterward, the change in each autonomic measure was calculated by subtracting the minimum value from the maximum in the time interval after the stimulus. This was done separately for each stimulus. Values above or below 2 standard deviations from the mean were considered outliers and were removed from the analysis.

### Statistical Analyses of the Autonomic Response During Language Evaluation

To analyze the effect that the different language subtests had on the mean and change (maximum – minimum) of EDA, HR, ABP, SBP and DBP, 10 repeated-measures ANOVAs with task (4 levels: B, YNQ, AWR, and VR) as within-subject factor, were conducted. Given that the number of items varied across tasks and in addition outliers and artifacts were removed, the total number of items included in each ANOVA analysis varied. The final *n* included in each analysis is specified in the result section. The general hypothesis was that the high-demanding language tasks (YNQ and AWR) would trigger a stronger emotional response than the low-demanding language task and this should be evidenced in the autonomic responses. Therefore, a trend analysis was performed to ascertain for quadratic non-linear relationship between the studied autonomic measures and the different conditions. Note that to perform a trend analysis, it is of utmost importance to enter the variables in a meaningful order. Thus, for all the reported ANOVA analyses, the different levels of the factor “task” were entered in the following order: B, YNQ, AWR, and VR. Keeping this order fixed allowed to the interpretation of potential significant quadratic effects positing that a given autonomic response increases from the baseline to the YNQ, then kept stable during the AWR, and then from the AWR to the VR task it decreases again. When a significant effect was found, Bonferroni *post hoc* multiple comparison test was applied to explore specific pair-wise differences between conditions. Statistical analysis was performed with IBM SPSS statistic software version 23.0.

### Neuroimaging Acquisition

MRI studies were performed on a 3-T MRI scanner (Philips Intera, Amsterdam, Netherlands), Release 3.2.3.4, with a MASTER gradient system (nominal maximum gradient strength = 30 mT/m, maximum slew rate = 150 mT/m/ms), equipped with a six-channel Philips SENSE head coil. Head movements were minimized using head pads and a forehead strap. High-resolution T-1 structural images of the whole brain were acquired with three-dimensional (3D) magnetization prepared rapid acquisition gradient echo (3D MPRAGE) sequence. Structural brain scans were acquired for the patient and for 25 healthy controls matched by age (mean age: 58 ± 5 years; range: 48–67 years) (Crawford *t*-test: *t* = 1.42, *p* = 0.16, two-tailed) and years of education (mean years of education: 18.9 ± 3.9 years; range: 10–25 years) (Crawford *t*-test: *t* = 1.26, *p* = 0.22, two-tailed). The acquisition parameters for the control group were: echo time (TE) about 4.6 ms; repetition time (TR) about 9.9 ms; acquisition matrix 240/200; field of view 240; turbo field echo (TFE) factor 200; flip angle 8°; reconstruction voxel size 1 × 0.94 × 0.94 mm; 190 contiguous slices; total acquisition time of the sequences about 170 s. The acquisition parameters for Mrs. A were as follows: echo time (TE) = 4.5 ms; repetition time (TR) = 9.8 ms; acquisition matrix 312/297; field of view 250; turbo field echo (TFE) factor 179; flip angle 8°; reconstruction voxel size 0.80 × 0.78 × 0.78 mm; 220 contiguous slices; total acquisition time of the sequence was 172 s.

Positron emission tomography (PET) data acquisitions were performed on a Discovery ST PET/CT camera (General Electric, Milwaukee, WI) after an intravenous injection of about 3.3 MBq/Kg for 25 healthy controls and 3.9 MBq/Kg for Mrs. A. PET images were reconstructed using 3D FORE-IR algorithm with CT attenuation correction (Matrix size, 128 × 128 × 47; voxel size, 2.34 × 2.34 × 3.27 mm).

Resting state functional MRI (rs-fMRI) was acquired while the patient was instructed to lie still with open eyes, without falling sleep. Two hundred volumes were collected with the following parameters: FFE/EPI sequence, TR = 3000 ms, TE = 35 ms, flip angle 82°, FOV = 220 mm, image matrix of 73/128 r, 50 slices with no gap and an acquisition voxel size of 1.72 mm × 1.72 mm × 3.00 mm.

### PET Statistical Analysis

Spatial preprocessing and statistical analysis were performed with Statistical Parametric Mapping (SPM12), running on MATLAB R2016b (Mathworks Inc., Natick, MA, United States). All T-1 structural images and 18F-FDG-PET images were manually aligned to anterior-posterior commissure (AC-PC) orientation. Reoriented PET images were then coregistered with T-1 images. A lesion mask, drawn over the T-1 image of the patient in native space, was applied to the T-1 image. Then, the coregistered volumes and the lesion mask were spatially normalized onto the MNI template (McGill University, Montreal, QC, Canada) using the T-1 images to calculate the deformation fields. The size of the resulting voxels was 2 × 2 × 2 mm. The normalized PET studies were smoothed with a FWHM 8-mm Gaussian kernel. Histogram-based intensity normalization was performed using in-house software. In this procedure, the smoothed images of each subject were voxel-wise divided by the mean of the normalized and smoothed images of the healthy controls. Histograms of these masked ratio images were generated, excluding damaged areas and ventricles. Finally, each original smoothed PET study was divided by the most prevalent value in its ratio image.

A region of interest (ROI) analysis was performed with the preprocessed PET studies. An average normalized activity concentration was calculated for each ROI and subject, excluding damaged areas from the ROIs of Mrs. A. Then a general linear model was applied for each ROI with sex and group (control or patient) as regressors. After estimating the coefficients with a robust fit, a *t*-test was applied for the group regressor, and the *z*-score for each ROI was then estimated. An absolute *z*-score ≥ 1.96 reflects a *p*-value ≤ 0.05; and a *z*-score ≥ 2.58 reflects a *p*-value ≤ 0.01, meaning that a given ROI shows significant differences in metabolism between the patient and the group of healthy controls. The ROIs included in the analysis were the amygdala, cingulate cortex (CC), OFC, HC, parahippocampal gyrus (PHC) and perisylvian areas (PSA) in both hemispheres. The PSA ROI comprised the rolandic operculum, subareas of the inferior frontal gyrus (IFG) [pars triangularis (pTr), pars opercularis (pOp) and pars orbitalis (pOr), precentral gyrus, postcentral gyrus, supramarginal gyrus (SMG), angular gyrus (AG), inferior parietal cortex, middle temporal gyrus (MTG), inferior temporal gyrus (ITG), superior temporal gyrus (STG), and the insula in both hemispheres]. All the ROIs were obtained from the AAL atlas as provided by the Wake Forest University (WFU) Pickatlas toolbox^2^ (Maldjian et al., 2003).

### Resting State Functional Connectivity Preprocessing and Analysis

Resting state image preprocessing was performed with Statistical Parametric Mapping software (SPM12)^3^ using a standard pipeline for resting state analysis. Functional data was AC-PC orientated, and the functional images were realigned to the first volume of the series. Coregistration between the mean functional and the structural T-1 was performed. Cost function masking was used during segmentation of the T-1 image in different tissues in order to disregard the damaged tissue when calculating the normalization transformations to be applied to the functional data normalization (Brett et al., 2001). For this purpose, a binary lesion mask was created by manually delineating the damaged areas directly on the T-1 image using MRIcron software (Rorden and Brett, 2000). The normalization parameters resulted from the T-1 segmentation were used for the normalization of the functional images to the standard MNI space. Finally, a smoothing with an 8-mm FWHM Gaussian kernel was applied to the functional images.

Exploratory seed-driven functional connectivity correlational analysis were performed with the CONN functional connectivity Toolbox v17^4^ (Whitfield-Gabrieli and Nieto-Castanon, 2012). After preprocessing, non-neuronal sources of noise were removed using CompCor method implemented in CONN toolbox. Specifically, the signal resulting from the voxels corresponding with the cerebrospinal fluid, the white matter and the brain lesion, as well as the motion parameters derived from the realignment preprocessing step, were removed with regression. A band-pass filter of 0.008 Hz – 0.09 Hz was applied to the residual BOLD time course to remove the effects of low frequency and high frequency noise outside our range of interest. ROI-based analysis was carried out by computing the temporal correlation between the BOLD signals from each voxel within the seed ROIs to each voxel within the target ROIs. For that, bi-variate correlations were performed between each pair of ROIs. ROIs were selected in base on their association to either language processing or anxiety/stress response. Specifically, the selected language-related ROIs were the IFG (pTr, pOp), insula, posterior STG (pSTG), posterior MTG (pMTG), SMG, and AG; whereas the selected *anxiety*-related ROIs were ACC, OFC, mPFC, amygdala, HC and PHC. All the ROIs were used as seed and as target ROIs in both hemispheres. For the rs-fMRI, we do not have a control sample, therefore only the single-level 1st level results are presented in this study. Correlations ≥ 0.35 are considered meaningful.

## Results

### Neuropsychological Findings

Tables 1, 2 show Mrs. A’s performance across all evaluations. Baseline evaluation scores reflect that Mrs. A had a severe language impairment affecting spontaneous speech, auditory comprehension and naming. Performance in the repetition subtest of the WAB was relative preserved compared to other linguistic domains. However, a more in depth evaluation with the PALPA and experimental subtests (Berthier et al., 2014a) showed that repetition of non-words and completion of open-ended sentences were markedly impaired, findings reflecting some involvement of the left PSA (Grossi et al., 1991). Reading comprehension was markedly impaired and oral reading showed the pattern of phonological dyslexia (mild impairment of word reading and marked impairment of non-word reading) (Crisp and Lambon Ralph, 2006).

The language testing made by the therapist revealed clinical improvement on the WAB-AQ score (6.6 pts in the WAB-AQ) under Rotigotine (8 mg/day) treatment (T1) with minimal point improvement in successive evaluations during continued treatment with the drug alone (T2: 0.9, T3: 0.7) and when ILAT was added (T4:1.1). Pharmacological treatment promoted improvements in spontaneous speech and auditory verbal comprehension, while naming stayed relatively stable throughout. Since performance in verbal repetition was almost at celling, no major changes were observed in this domain. Surprisingly, although Mrs. A’s relatives reported that she had improved substantially with both interventions, no further improvements were observed after combined therapy (Rotigotine-ILAT) when she was assessed by the therapist. However, when language evaluation was performed by her husband (previously trained), significant improvement in the severity of aphasia (WAB-AQ) was seen. In fact, a difference of 12.1 points in the WAB-AQ was observed when comparing the evaluations of the therapist and the husband, having better outcomes in the last one (*p* = 0.057, Fisher exact test, one tailed) (see Table 2). Mrs. A received around 400 h of ILAT in 140 days.

### Neuropsychiatric Findings

At baseline, a high agreement in LOI items evaluating excessive perfectionism, self-demanding, and methodical style was found when comparing the answers of Mrs. A’ husband and son (see Table 3). Some items in which they agreed answering “yes” were: “Are you the sort of person who has to pay a great deal of attention to details?”, “Are you ever over-conscientious or very strict with yourself?”; “Are you very systematic and methodical in your daily life?”; “Do you like to get things done exactly right, down to the smallest detail?”; “Do you think it is important to follow rules and regulations exactly?”. Note that both relatives were instructed to answer by taken in consideration the premorbid symptoms and traits of Mrs. A. The CRS completed by the therapist during observation of Mrs. A’s behaviour yielded a score of 8 points, just approaching the abnormal scores (Starkstein et al., 1993). High scores were rated in the following items: “patient appeared to be anxious”, “complained of feeling anxious or afraid” (scoring 3 in both indicating severe symptomatology) and “patient refused to do something” (scoring 2 indicating moderate symptomatology).

**Table 3:** Results of the ROI analysis for the PET studies.

	**Left**	**Right**
Amygdala	-4.59	-0.15
Cingulate Cortex	-6.29	-4.05
Orbitofrontal Cortex	-5.29	-0.22
Hippocampus	-6.22	0.15
Parahippocampus	-5.93	0.86
Perisylvian Area	-6.36	0.65


*The *z*-scores for the comparison between Mrs. A and the control group are shown. Values over 1.96 or below -1.96 reflect *p*-values below 0.05; and values higher than 2.58 or smaller than -2.58 reflect *p*-values below 0.01.*

### Autonomic Response

**FIGURE 3:**
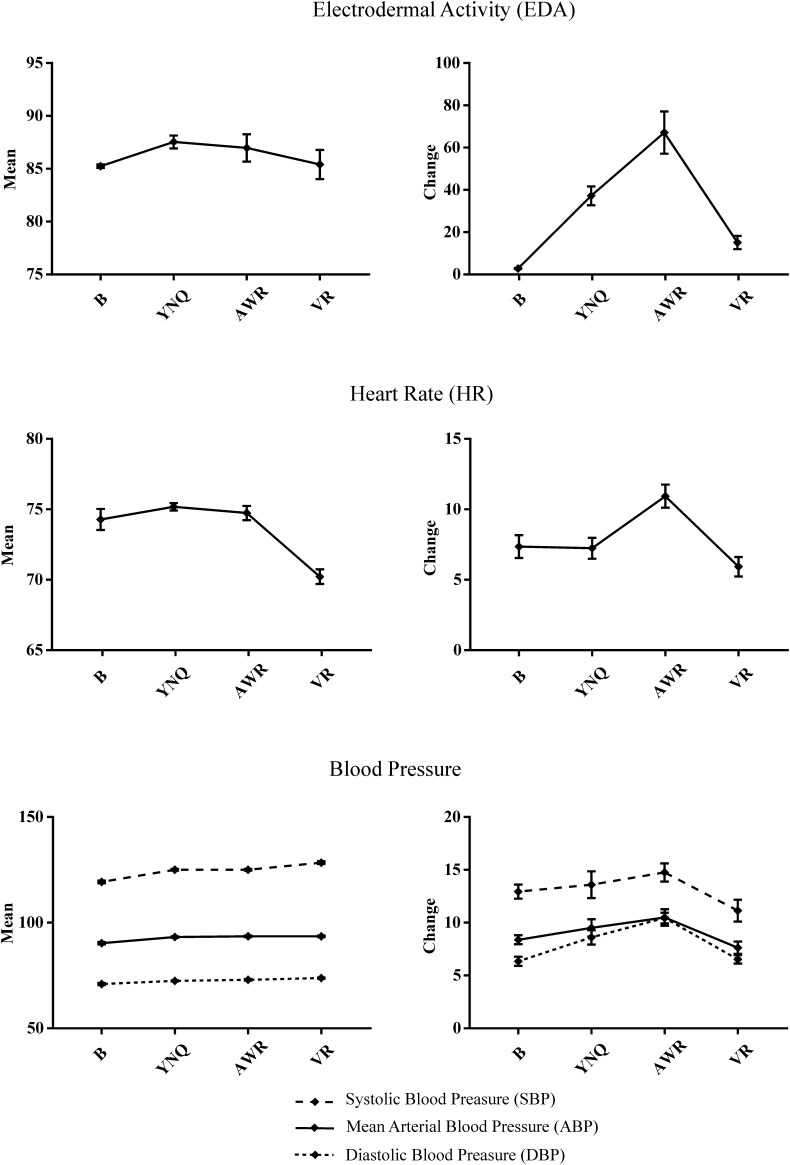
Plots showing the patterns of the different autonomic responses in three different linguistic subtests of the Western Aphasia Battery (WAB): Yes/No Questions (YNQ); Auditory Word recognition (AWR); Verbal Repetition: VR. These tasks entailed high (YNQ and AWR) or low (VR) difficulty for the patient which resulted from the type and severity of the aphasia profile. The range of possible changes in mean activity for each task is represented in the orderly, whereas baseline (B) and the three language tasks are shown in abscissa. Note that AWR has, in addition, a motor component (patient had to point to the correct picture) that may further increase autonomic reactivity. For each task, mean and error bars are plotted. Error bars represent the standard error of the mean (SEM).

Figure 3 shows the results of the autonomic response analysis. Repeated-measures ANOVA analysis for mean EDA revealed a significant quadratic trend [*F*(1,12) = 5.51, *p* = 0.037, η^2^= 0.31]. No main effect of task was found in mean EDA (*p* = 0.21). *Post hoc* tests using Bonferroni correction revealed that mean EDA level in YNQ was higher than in B (*p* = 0.018), whereas no other significant differences were found in pair-wise comparisons. The same ANOVA analysis was performed with EDA change (maximum value – minimum value, calculated for each trial independently), revealing a main effect of task [F(3,33) = 26,67, *p* = 0.000, η^2^= 0.7], and a significant linear [*F*(1,11) = 18.29, *p* = 0.001, η^2^= 0.62] and quadratic [*F*(1,11) = 56.43, *p* = 0.000, η^2^= 0.83] trend. *Post hoc* tests revealed that EDA change in B was lower compared to the change in YNQ (*p* = 0.000), AWR (*p* = 0.000) and VR (*p* = 0.014); and that EDA change in VR was lower compared to YNQ (*p* = 0.013) and AWR (*p* = 0.001), indicating stronger autonomic reactivity in these two last tasks.

There was a significant main effect of task in mean HR response [F(3,39) = 17.85, *p* = 0.000, η^2^= 0.59]. Both a significant linear [*F*(1,13) = 25.03, *p* = 0.000, η^2^= 0.65] and a quadratic [*F*(1,11) = 33.26, *p* = 0.000, η^2^= 0.71] trends were found. *Post hoc* tests showed that the mean HR response was lower during VR task compared to B (*p* = 0.007), YNQ (*p* = 0.000) and AWR (*p* = 0.001). ANOVA analysis on HR change disclosed a main effect of task [*F*(3,39) = 7.86, *p* = 0.000, η^2^= 0.37], and a significant quadratic effect [*F*(1,13) = 9.98, *p* = 0.008, η^2^= 0.43]. *Post hoc* tests evidenced that the HR change during AWR was higher than during both YNQ (*p* = 0.031) and VR (*p* = 0.002).

In reference to the mean ABP, the repeated-measured ANOVA disclosed a main effect of task [*F*(3,39) = 19.33, *p* = 0.000, η^2^= 0.59] and a linear trend [*F*(1,13) = 37, 37, *p* = 0.000, η^2^= 0.74]. No quadratic effect was found. *Post hoc* comparisons revealed that mean ABP in B was lower than in YNQ (*p* = 0.008), AWR (*p* = 0.001) and VR (*p* = 0.000). For ABP change, a main effect of task [*F*(3,33) = 4.45, *p* = 0.01, η^2^= 0.28] and a quadratic trend [*F*(1,11) = 12. 83, *p* = 0.004, η^2^= 0.53] were found. *Post hoc* comparisons disclosed that ABP change during VR was lower than during AWR (*p* = 0.019).

Repeated-measured ANOVA analysis of mean SBP revealed a main effect of task [*F*(3,39) = 43.93, *p* = 0.000, η^2^= 0.77] and a linear [*F*(1,13) = 61.58, *p* = 0.000, η^2^= 0.82] and quadratic [*F*(1,13) = 5. 09, *p* = 0.042, η^2^= 0.28] trends. *Post hoc* tests showed that mean SBP was lower in B compared to YNQ (*p* = 0.000), AWR (*p* = 0.000) and VR (*p* = 0.000); and that mean SBP was higher in VR compared to YNQ (*p* = 0.026) and AWR (*p* = 0.003). For SBP change, a main effect of task [*F*(3,33) = 3.11, *p* = 0.039, η^2^= 0.22] and a quadratic effect [*F*(1,11) = 4.98, *p* = 0.047, η^2^= 0.31] were found. Pos-hoc comparisons disclosed that SBP change was lower during B than during AWR (*p* = 0.013).

Finally, referred to mean DBP, ANOVA analysis revealed a main effect of task [*F*(3,39) = 10.29, *p* = 0.000, η^2^= 0.44] and a linear effect [*F*(1,13) = 21.41, *p* = 0.000, η^2^= 0.62]. *Post hoc* tests showed that mean DBP was lower in B than in AWR (*p* = 0.004) and in VR (*p* = 0.006). For DBP, a main effect of task [*F*(3, 36) = 15.99, *p* = 0.000, η^2^= 0.57] and a quadratic effect [*F*(1,12) = 35.33, *p* = 0.000, η^2^= 0.74] were found. *Post hoc* tests disclosed that DBP change was lower both during B (*p* = 0.000) and VR (*p* = 0.000) compared to AWR.

### Neuroimaging Results

#### Positron Emission Tomography Results

ROI analysis in PET revealed significant hypometabolic activity in the left Amygdala, CC, OFC, HC and some components of the PSA (*p* = 0.01). In the right hemisphere, significant hypometabolic activity was seen only for the CC. No statistical significant areas of hypermetabolism were found in any of the ROIs; however, higher activation than the mean activation of the control group was found in the right PSA (*z*-score = 0.65) and right parahippocampus (0.86) (see Table 3).

#### Resting State Functional Connectivity Results

**FIGURE 4:**
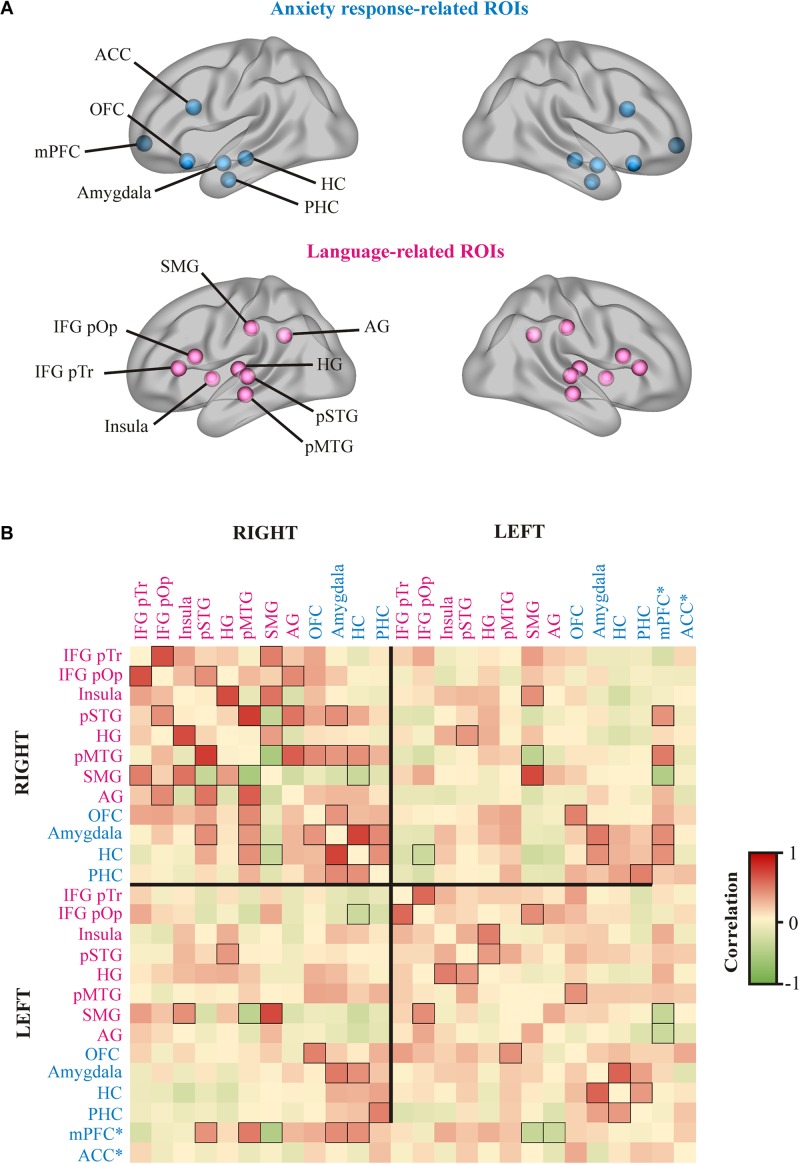
Resting state functional connectivity results. **(A)** Regions of interest (ROI) used for functional connectivity analyses. Blue balls represent the ROIs associated to the anxiety response circuits, whereas pink balls represent ROIs related to language processing. **(B)** Heat map representing the matrix of bi-variate correlations values between each pair of ROIs for both hemispheres. The scale color bar located at the right side of the figure indicates the degree of connectivity. The boxes representing a correlation greater than 0.35 or smaller than –0.35 show the edges highlighted in black. ^∗∗^ mPFC and ACC were bilateral ROIs. ACC, anterior cingulate cortex; OFC, orbitofrontal cortex; mPFC, medial prefrontal cortex; HC, hippocampus; PHC, parahippocampus; SMG, supramarginal gyrus; AG, angular gyrus; IFG pOp, inferior frontal gyrus pars opercularis; IFG pTr, inferior frontal gryus pars triangularis; HG, Heschl’s gyrus; pSTG, posterior superior temporal gyrus; pMTG, posterior middle temporal gyrus.

The resulting functional connectivity matrix reflects the bivariate correlation between each pair of ROIs (Figure 4A). This matrix is plotted as a heat-map (warmer color represents larger positive correlations whereas green color represents larger negative correlations) (see Figure 4B). Functional connectivity at single level reveals a set of regions showing a trend to be more functionally coupled. Only the patterns considered more meaningful for the current study were highlighted here. As a general pattern, there was an increased connectivity among the ROIs within the right hemisphere. Specifically, within the subset of language-related ROIs and within the subset of anxiety response-related ROIs. In addition, the right OFC showed a stronger coupling with the bilateral Amygdala and the right pMTG, the Amygdala and HC showed stronger functional synchronization with regions within the anxiety network in the right hemisphere and posterior temporo-parietal PSA. Interestingly, the mPFC was highly coupled with the right language-related PSA (pSTG, pMTG) but it did not show high correlations with the left PSA.

## Discussion

In the present study we reported the case of Mrs. A, a woman who had a severe chronic MTCA and overwhelming LA after suffering two simultaneous left hemisphere hemorrhages. Clinical observations during language testing suggested that evaluation contexts induced anxiety and catastrophic reactions in Mrs. A, which in turn intensified automatic echolalia and perseverative responses (e.g., “No, no, no…”, “I can’t, I can’t, I can’t”). In fact, both repetitive verbal behaviors were the most evident linguistic manifestations of LA. For the study of Mrs. A, we used an integrative approach that included examination of language and cognitive functions, premorbid personality traits and autonomic response induced by linguistic tasks as well as the exploration of the neural basis of LA using multimodal neuroimaging.

Since there are not suitable verbal self-reports of LA in persons with severe aphasia, its detection becomes difficult in this population (Morris et al., 2017). Therefore, identifying linguistic and non-linguistic warning signs that may transpire discomfort and anxiety has an important clinical value. In fact, LA has detrimental consequences on linguistic performance (Cahana-Amitay et al., 2015) and its presence may guide clinical decisions in the wrong direction. In line with previous studies (Buchanan et al., 2014; Cahana-Amitay et al., 2015), the data obtained in the present work patently shows that the performance of Mrs. A in standardized aphasia batteries (e.g., WAB) was worse when language testing was performed by a therapist (stressful situation) than when assessment was performed by a relative at home. The first evaluation (T1), performed in a clinical setting after 1 month of Rotigotine treatment (8 mg/day), revealed clinical improvement (>5 points in the WAB-AQ) indicating that Mrs. A was a “responder” to this intervention (Berthier et al., 2009). This suggests that the powerful effect of the initial dopaminergic stimulation with Rotigotine attenuated, at least partially, the negative impact of LA in language testing. Nevertheless, only minimal improvements in formal language evaluations performed by the therapist were found thereafter with Rotigotine alone at the same dose (T2 and T3) as well as during combined treatment with Rotigotine and ILAT (T4). It was noteworthy that when language evaluation (T4) was administered by Mrs. A’s husband, the WAB-AQ yielded significantly higher scores, increasing the score by 12.1 points in comparison with the evaluation performed by the therapist. This suggests that LA had a negative impact on Mrs. A’s linguistic performance in clinical settings, masking treatment-induced improvements after prolonged treatment with Rotigotine and very intensive use of ILAT (∼400 h in 140 days!).

**FIGURE 5:**
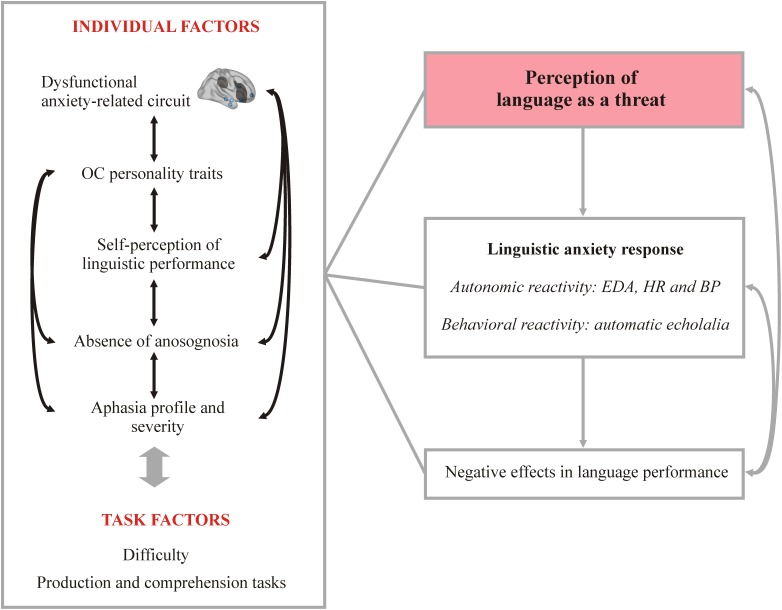
Model proposed to describe the linguistic anxiety response in Mrs. A. The schema depicts different individual and task-related factors that may interact among them, resulting ultimately in the perception of language as a threat, triggering the stress response and negatively modulating language performance. OC, obsessive–compulsive; EDA, electrodermal activity; HR, heart rate; BP, blood pressure.

We documented that several factors may have contributed to Mrs. A‘s negative appraisal of language in testing contexts increasing the distortion of her language behavior (i.e., worsened performance and increased echolalia and perseverations). These key factors are described in turn (see Figure 5). First, the identified premorbid obsessional personality style of Mrs. A (i.e., perfectionism and self-criticism of potential failures) most likely contributed to increment her anxious reactions during language testing. Second, the severity of aphasia in Mrs. A, with almost inexistent spontaneous speech (Parr, 2007; Thomas and Lincoln, 2008) together with a preserved awareness of her linguistic deficit may have also favored the strong feeling of low self-efficacy in language performance, thus heightening LA (Bandura, 1988). Lastly, the direct and remote effects of the lesion may also have an important role since key areas involved in the subjective appraisal of stressors and in the regulation of the associated autonomic and behavioral consequences were affected.

The analysis of Mr A’s autonomic reactivity in response to different language tasks under Rotigotine treatment (T2) showed a greater EDA change and mean HR during linguistic tasks for which she had greater difficulty (i.e., YNQ and AWR) than in tasks that evaluated a preserved linguistic domain (i.e., VR). Specifically, both EDA change and mean HR across YNQ and AWR were greater compared to the same indexes measured during VR. In fact, EDA change for VR was not different to EDA change observed during baseline recording. The greater change in the AWR should be taken with care since it could be boosted by the motor component of this task in which Mrs. A had to point to the picture named by the examiner. In contrast, the YNQ does not have a motor component; therefore, greater reactivity in this task is likely to be associated with the anxiety response evoked by the challenging language context. We did not find the expected pattern in either mean or blood pressure change (ABP, SBP and DBP) or HR change. Thus, the autonomic study of Mrs. A revealed the expected pattern for some measures (i.e., EDA) but not for others (i.e., ABP, SBP, DBP).

Significant changes in blood pressure and HR in subjects exposed to stressful situations have been reported in brain damaged and healthy subjects. For instance, a previous study of anxiety in a person with acquired apraxia of speech reported more striking changes in ABP (SBP and DBP) and HR when therapy was performed by a therapist (stressful context) than during a self-administered therapy (Sakamoto et al., 1999). Experimental studies inducing anxiety in healthy subjects showed greater autonomic reactivity than the observed in Mrs. A. For instance, Gonzalez-Bono et al. (2002) reported an average HR increase of > 10 beats/minute in participants expressing low cognitive anxiety but > 20 beats/minute in high anxiety subjects when giving a public speech. In any case, this leaves open the possibility that the autonomic response of subjects like Mrs. A confronting with stressful situations may differ from the response found in healthy subjects and in persons with brain lesions that did not affect the cortical-subcortical network regulating autonomic response. Here, it is possible that the blunted blood pressure and heart rate response to tasks manipulation observed in Mrs. A might be a consequence of extensive direct damage as well as the remote structural, metabolic and functional connectivity changes induced by the brain lesions (diaschisis) (Carrera and Tononi, 2014). Abnormal cardiovascular response in patients with focal damage to the anterior CC confronting with cognitive stressful situations has been previously reported (Buchanan et al., 2010).

Understanding the nature of LA response and its negative effects in Mrs. A’s language performance, requires considering that the structural and functional metabolic changes, besides affecting some components of the left PSA, additionally involved limbic and paralimbic areas rarely damaged in PWA (Kreisler et al., 2000; Fridriksson et al., 2018) (see Table 3). Studies of the neural basis of anxiety in healthy population have provided knowledge on the functional complexity of the brain cortical-subcortical networks that participate in emotional and cognitive control. The ACC, OFC and mPFC have been pinpointed as relevant areas for (1) the appraisal and expression (behavioral and autonomic) of emotions; (2) control of inhibitory processes; and (3) the regulation of cognitive processes (Etkin and Wager, 2007; Stevens et al., 2011). Moreover, the mPFC regulates the HR response to social threats in a complex manner since different regions have a completely different role, sometimes modulating opposite effects (Wager et al., 2009). Further, these areas are strongly interconnected to the amygdala (affected in Mrs. A) and exert a regulatory effect over it, modulating emotional responses (Kim et al., 2011; Motzkin et al., 2015). Importantly, structural, functional and metabolic findings of Mrs. A showed involvement of several areas of this cortico-subcortical network. The PET analysis revealed decreased metabolic activity in the left amygdala, left OFC and bilateral CC. Further, resting state functional connectivity analyses suggested a functional decoupling between the left amygdala and both the mPFC and the OFC, whereas the right amygdala seemed to be synchronized with regions from the PSA, the mPFC and the OFC in the right hemisphere. The fact that preserved sectors of the left PSA were functionally disconnected from the mPFC suggests that the release of automatic echolalia as a manifestation of LA resulted from the disinhibition of the left PSA (Berthier et al., 2018). More support for this possibility comes from the PET finding showing decreased metabolic activity in the left OFC. Therefore, despite that the OFC is functionally connected to the pMTG, this may not exert the required control over the activity of the left PSA, contributing to the increment of automatic echolalia under stressful conditions. Dysfunctional activity in the network formed by the amygdala and prefrontal areas could also play a role at this stage. Since mPFC areas are involved in both cognitive control and the regulation of emotional responses, demanding linguistic tasks may require higher cognitive control, modifying the activity of frontal areas, and at the same time affecting the emotional response to these tasks through cortico-subcortical connections. Although the current evidence suggests that this network was dysfunctional in Mrs. A, further studies are needed to gain more insight on the brain correlates of LA.

## Conclusion

Anxiety is a frequent aftermath of stroke. In PWA, anxiety induced by language testing and everyday situations that require the use of language is challenging since it affects linguistic performance, jeopardizing the reliability of evaluations outcome. Intriguingly, there are very few studies aiming to explore the clinical features and neural correlates of LA. The present work provides a detailed multimodal characterization of a person with severe aphasia and LA under different interventions and assessed in different contexts. The results from this study provide further evidence on the detrimental effects of LA in language use, confirming poorest language performance when the evaluation was performed by a therapist (stressful context) compared to testing performed by a well-trained relative. In addition, we identified some factors (e.g., premorbid personality traits) that may increase LA, the underlying neural mechanisms and its detrimental effect on language performance. Further longitudinal studies with larger sample of participants are needed to advance in the characterization of LA and to help to design more optimal protocols for PWA.

## Author Contributions

All authors listed have made a substantial, direct and intellectual contribution to the work, and approved it for publication. All authors were involved in conception and design, acquisition of data, or analysis and interpretation of data. MJT-P, DL-B, and MB were directly implicated in performing language, and cognitive testing, as well as training the patient’s husband in the application of speech therapy and language assessment. MJT-P, DL-B, JP-P, NR-V, and MB analyzed and/or interpreted neuroimaging data. MJT-P, DL-B, and MD-M participated in the acquisition, statistical analysis, and/or interpretation of autonomic data. All authors drafted the article and revised it critically for important intellectual content.

## Conflict of Interest Statement

The authors declare that the research was conducted in the absence of any commercial or financial relationships that could be construed as a potential conflict of interest.

## Footnotes

1 Note that obsessive–compulsive disorder was considered an anxiety disorder until the last version of the Diagnostic and Statistical Manual of Mental Disorders (DSM-5) (American Psychiatric Association, 2013).

2 http://www.fmri.wfubmc.edu/cms/software

3 http://www.fil.ion.ucl.ac.uk/spm/

4 http://www.nitrc.org/projects/conn
